# Impaired B Cell Function in Mice Lacking Perforin-2

**DOI:** 10.3389/fimmu.2020.00328

**Published:** 2020-02-27

**Authors:** Daniela Frasca, Alain Diaz, Maria Romero, Thomas Vazquez, Natasa Strbo, Laura Romero, Ryan M. McCormack, Eckhard R. Podack, Bonnie B. Blomberg

**Affiliations:** Department of Microbiology and Immunology, University of Miami Miller School of Medicine, Miami, FL, United States

**Keywords:** splenic B cells, adipose tissue B cells, inflammation, perforin-2, antibody responses

## Abstract

Perforin-2 (P2) is a pore-forming protein with cytotoxic activity against intracellular bacterial pathogens. P2 knockout (P2KO) mice are unable to control infections and die from normally non-lethal bacterial infections. Here we show that P2KO mice as compared to WT mice show significantly higher levels of systemic inflammation, measured by inflammatory markers in serum, due to continuous microbial translocation from the gut which cannot be controlled as these mice lack P2. Systemic inflammation in young and old P2KO mice induces intrinsic B cell inflammation. Systemic and B cell intrinsic inflammation are negatively associated with *in vivo* and *in vitro* antibody responses. Chronic inflammation leads to class switch recombination defects, which are at least in part responsible for the reduced *in vivo* and *in vitro* antibody responses in young and old P2KO vs. WT mice. These defects include the reduced expression of activation-induced cytidine deaminase (AID), the enzyme for class switch recombination, somatic hypermutation and IgG production and of its transcriptional activators E47 and Pax5. Of note, the response of young P2KO mice is not different from the one observed in old WT mice, suggesting that the chronic inflammatory status of mice lacking P2 may accelerate, or be equivalent, to that seen in old mice. The inflammatory status of the splenic B cells is associated with increased frequencies and numbers of the pro-inflammatory B cell subset called Age-associated B Cells (ABCs) in the spleen and the visceral adipose tissue (VAT) of P2KO old mice. We show that B cells differentiate into ABCs in the VAT following interaction with the adipocytes and their products, and this occurs more in the VAT of P2KO mice as compared to WT controls. This is to our knowledge the first study on B cell function and antibody responses in mice lacking P2.

## Introduction

Perforin-2 (MPEG1, P2) is a pore-forming protein with broad-spectrum activity against infectious bacteria in both mice and humans (1). P2 is constitutively expressed in phagocytes and other immune cells and can be induced in parenchymal, tissue-forming cells (2, 3). *In vitro*, P2 prevents the intracellular replication of bacterial pathogens (3). P2 knockout (P2KO) mice are unable to control the systemic dissemination of bacterial pathogens and die from bacterial infections that are normally not lethal (3). Other bactericidal molecules have been found to be less effective in the absence of P2, suggesting that P2 is essential for the activity of mammalian immune defense mechanisms. It has recently been shown that P2 facilitates the delivery of proteases and other antimicrobial effectors to the sites of bacterial infection leading to effective killing of phagocytosed bacteria (4).

Translocation of bacteria and their products from the gastrointestinal tract to extra-intestinal sites (lymph nodes, liver, spleen, kidney, blood) is a phenomenon that may occur spontaneously in healthy conditions in humans and mice without apparent deleterious consequences (5). Bacterial translocation is increased in different clinical pathological conditions and is certainly involved in the pathophysiological mechanisms of many diseases. Translocation of bacteria and/or their toxic products from the gastro-intestinal tract is strongly suspected to be responsible for the establishment of systemic chronic inflammation. This condition may be exacerbated in P2KO mice.

We have previously shown in mice (6) and humans (7, 8) that B cell function decreases with age and this decrease is associated with chronic low-grade systemic inflammation, called “inflammaging” (9). Higher levels of inflammaging, measured by serum TNF-α, induce higher TNF-α production by B cells from old mice and humans *in vivo* and *in vitro*, leading to significant decreases in their capacity to make protective antibodies in response to antigenic/mitogenic stimulation (6, 7). Serum TNF-α has been shown to up-regulate the expression of its receptors (TNFRI and TNFRII) on B cells, and interaction of TNF-α with its receptors induces NF-kB activation and secretion of TNF-α as well as of other pro-inflammatory cytokines and chemokines (10). Importantly, blocking TNF-α with specific antibodies has been shown to increase B cell function, at least *in vitro*, in both mice (6) and humans (7).

The purpose of this study is to evaluate B cell function in P2KO mice. We hypothesized that P2KO mice are unable to control the translocation of bacteria and/or toxic bacterial products and this would generate a systemic low-grade chronic inflammation which negatively affects B cell function and antibody responses. Our results herein show that this is indeed the case. P2KO mice show significantly higher levels of systemic and intrinsic B cell inflammation which are negatively associated with protective antibody responses to a vaccine. This is to our knowledge the first study evaluating B cell function and antibody responses in mice lacking P2.

## Materials and Methods

### Mice

Male P2KO and wild type (WT) mice, both on a 129/SvJ background, were generated as previously described (3). Mice were young (3–4 months) and old (>18 months), bred at the University of Miami, Miller School of Medicine Transgenic Core Facility. Mice were allowed to freely access food and water and were housed at 23°C on a 12 hr light/dark cycle under specific pathogen-free conditions. All studies adhered to the principles of laboratory animal care guidelines and were IACUC approved (protocols #16-252 and #16-006).

### Influenza Vaccine Response

Mice were injected intramuscularly with 4 μg of the quadrivalent influenza vaccine (Fluzone Sanofi Pasteur 2017–2018) in alum (Aluminum Potassium Sulfate Dodecahydrate, SIGMA A-7210). Total volume of injection was 100 μl. Mice were sacrificed 28 days after the injection (peak of the response).

### B Cell Enrichment

B cells were isolated from the spleens after 20 min incubation at 4°C using CD19 MicroBeads (Miltenyi Biotec 130-121-301), according to the MiniMACS protocol (20 μl Microbeads + 80 μl PBS, for 10^7^ cells). At the end of the purification procedure, cells were 90–95% CD19-positive by cytofluorimetric analysis. They were then maintained in PBS for 3 hrs at 4°C to minimize potential effects of anti-CD19 antibodies on B cell activation. After positive selection, B cells were divided in two aliquots: one aliquot was used for culture stimulation, the other aliquot for RNA extraction after cells were resuspended in TRIzol (ThermoFisher Scientific).

### B Cell Culture

Splenic B cells (10^6^/ml) were cultured in complete medium (RPMI 1640, supplemented with 10% FCS, 100 U/ml Penicillin-Streptomycin, 2 × 10^−5^ M 2-ME, and 2 mM L-glutamine). FCS was certified to be endotoxin-free. B cells were stimulated in 24 well culture plates with 1 μg/ml of LPS (from *E. coli*, SIGMA L2880) for 1–7 days. At the end of the stimulation time, B cells were counted in a solution of trypan blue to evaluate viability which was found comparable in cultures of WT and P2KO mice.

### Isolation of Epididymal VAT

Epididymal VAT was collected, weighed, washed with 1X Hanks' balanced salt solution (HBSS), resuspended in Dulbecco's modified Eagle Medium (DMEM), minced into small pieces, passed through a 70 μm filter and digested with collagenase type I (SIGMA C-9263) for 1 hr at 37°C in a water bath. Digested cells were passed through a 300 μm filter, centrifuged at 300 g in order to separate the floating adipocytes from the stromal vascular fraction (SVF) containing the immune cells. The cells floating on the top were transferred to a new tube as adipocytes. The cell pellet (SVF) on the bottom was resuspended in a solution of Ammonium Chloride Potassium (ACK) for 3 min at RT (room temperature) to lyse the red blood cells. Both adipocytes and SVF were washed 3 times with DMEM. B cells were isolated from the SVF as indicated immediately below. Adipocytes were sonicated for cell disruption in the presence of TRIzol, and then centrifuged at 1,000 × g at 4°C for 20 min to separate the soluble fraction from the lipids and cell debris. The soluble fraction was then used for RNA isolation.

### Cell Staining

Splenic and VAT B cell subsets were identified by the following membrane markers. Follicular (FO): CD19+AA4.1-CD43-CD23+CD21^int^; Marginal Zone (MZ): CD19+AA4.1-CD43-CD23^low/neg^CD21^hi^; Age-associated B cells (ABC): CD19+CD43-AA4.1-CD23-CD21-. AA4.1 (CD93) is the marker of transitional B cells. CD43 is the marker of B1 B cells. Both transitional and B1 B cells are excluded by cell sorting to obtain only B2 B cells. Plasma cells were evaluated by membrane expression of CD138 in cultured cells.

B cells were membrane stained for 20 min at rt with Live/Dead detection kit (ThermoFisher) and with the following antibodies: PacBlue-conjugated anti-CD45 (Biolegend 103125), APC-Cy7-conjugated anti-CD19 (BD 557655), PE-Cy7-conjugated anti-AA4.1 (eBioscience 25-5892-81), APC-conjugated anti-CD43 (BD 560663), FITC-conjugated anti-CD21/CD35 (BD 553818), and PE-conjugated anti-CD23 (BD 553139). To evaluate plasma cell frequencies, splenic B cells were membrane stained with PE-conjugated anti-CD138 antibody (BD 553714). Cells were then fixed with BD Cytofix (BD 554655). Up to 10^5^ events in the lymphocyte gate were acquired on an LSR-Fortessa (BD) and analyzed using FlowJo 10.0.6 software.

### Cell Sorting

FO B cells were sorted with the Sony SH800 cell sorter. FO B cells were incubated with adipocytes in transwell as detailed below.

### RNA Extraction and cDNA Preparation

The mRNA was extracted from LPS-stimulated B cells at day 1 (to evaluate E47, Pax5) and at day 5 (to evaluate AID), using the μMACS mRNA isolation kit (Miltenyi), according to the manufacturer's protocol, eluted into 75 μl of preheated elution buffer, and stored at −80°C until use. Total RNA was extracted from unstimulated VAT B cells, as well as from adipocytes, resuspended in TRIzol, according to the manufacturer's protocol, eluted into 10 μl of preheated H_2_O, and stored at −80°C until use. Reverse Transcriptase (RT) reactions were performed in a Mastercycler Eppendorf Thermocycler to obtain cDNA. Briefly, 10 μl of mRNA or 2 μl of RNA at the concentration of 0.5 μg/μl were used as template for cDNA synthesis in the RT reaction. Conditions were: 40 min at 42°C and 5 min at 65°C.

### Quantitative PCR (qPCR)

Reactions were conducted in MicroAmp 96-well plates, and run in the ABI 7300 machine. Calculations were made with ABI software. Briefly, we determined the cycle number at which transcripts reached a significant threshold (Ct). A value for the amount of the target gene, relative to GAPDH, was calculated and expressed as ΔCt. Results are expressed as 2^−ΔΔCt^. Reagents and primers for qPCR amplification were from ThermoFisher. Primers were: GAPDH Mm99999915_g1, Tcfe2a/E47 Mm01175588_m1, Pax5 Mm00435501_m1, AID Mm00507774_m1, Prdm1 Mm00476128_m1, TNF-α Mm00443258_m1, IL-6 Mm00446190_m1, CXCL10 Mm00445235_m1, CCL2 Mm00441242_m1, CCL5 Mm01302427.

### Enzyme-Linked Immunosorbent Assay (ELISA)

To measure microbial translocation in serum, Lonza QCL-1000 kit was used for the detection of Gram-negative bacterial endotoxin.

To measure influenza vaccine serum IgG and IgA responses, the influenza vaccine was used for coating ELISA plates. The vaccine was used at the concentration of 10 μg/ml. Detection antibodies were HRP-conjugated affinity-purified F(ab')_2_ of a goat anti-mouse IgG (Jackson IR Labs 115-036-062) and HRP-conjugated goat anti-mouse IgA (ThermoFisher 62-6720).

To measure stool-specific IgG antibodies in serum, we first obtained total protein lysates from stools of WT and P2KO mice that were used for coating ELISA plates. Stool sample collection and processing was performed as described (11). Total protein lysates were obtained using the M-PER mammalian protein extraction reagent (ThermoFiscer 78501), according to the manufacturer's protocol. Protein lysates were used at the concentration of 10 μg/ml. Detection antibody was an HRP-conjugated affinity-purified F(ab')_2_ of a goat anti-mouse IgG (Jackson IR Labs 115-036-062).

To measure LPS-induced IgG3 in culture supernatants, purified IgG3 subclass-specific antibodies were used for coating (Southern Biotech 1101-01), at the concentration of 2 μg/ml. Detection antibody was the same as above.

### Co-culture of Adipocytes and Splenocytes

The ratio between adipocytes and splenic lymphocytes in co-cultures was equal to that which we measured in *ex vivo* isolated VAT (ratio adipocytes:lymphocytes). In the transwells, cells were co-cultured by using inserts with a 0.4 μm porous membrane (Corning) to separate adipocytes and splenic lymphocytes. Cells were left unstimulated. After 72 h, cells in the upper wells (splenic lymphocytes) were harvested, washed and stained to evaluate percentages and numbers of B cell subsets.

### Statistical Analyses

To examine differences between 4 groups, two-way ANOVA was used. Group-wise differences were analyzed afterwards with Bonferroni's multiple comparisons test, with *p* < 0.05 set as criterion for significance. To examine differences between 2 groups, Student's *t*-tests (two-tailed) were used. To examine the relationships between variables, bivariate Pearson's correlation analyses were performed, using GraphPad Prism 5 software. Principal Component Analyses (PCA) were generated using RStudio Version 1.1.463.

## Results

### Increased Microbial Translocation in the Serum of P2KO vs. WT Mice

**Figure 1:**
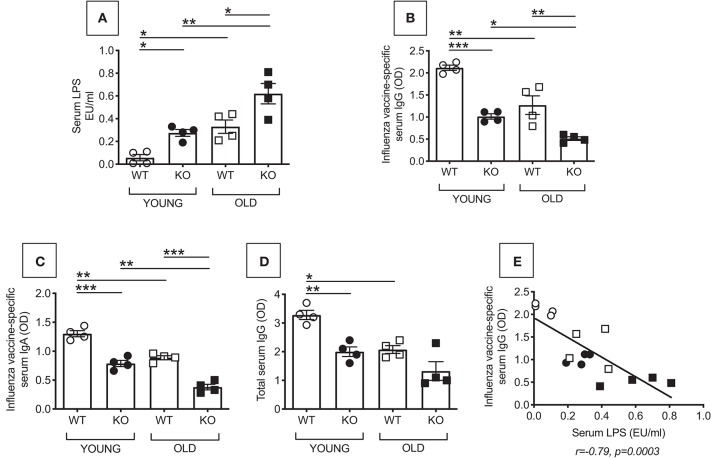
Increased microbial translocation and reduced *in vivo* influenza vaccine antibody response in P2KO vs. WT mice. **(A)** Microbial translocation in serum was measured by ELISA for LPS in young and old WT and P2KO mice (4 mice/group). Mean comparisons between groups were performed by two-way ANOVA. *p < 0.05, **p < 0.01. **(B)** To measure the influenza vaccine response, mice were immunized intramuscularly with the influenza vaccine. Serum response to the vaccine was evaluated at day 28 post vaccination by ELISA. **(C)** Influenza vaccine-specific IgA responses measured by ELISA at day 28 post vaccination. **(D)** Total serum IgG measured by ELISA. Mean comparisons between groups were performed by two-way ANOVA followed by Bonferroni's multiple comparisons test. *p < 0.05, **p < 0.01, ***p < 0.001. **(E)** Correlation of microbial translocation and influenza vaccine response. Pearson's *r* and *p*-values are at the bottom of the figure.

We first measured microbial translocation by quantifying serum levels of LPS, the major component of Gram-negative bacterial cell walls. LPS in serum indicates microbial translocation (12). Results in Figure 1A show increased serum LPS in young and old P2KO mice as compared to WT controls, the highest levels being observed in old P2KO mice. Serum LPS levels in young P2KO mice are comparable to those observed in old WT mice. These results confirm our initial hypothesis that translocation of bacteria and their products from the gastro-intestinal tract occurs in P2KO mice and this may be responsible for the establishment of systemic chronic inflammation. We have also measured bacterial translocation by serum levels of IgG antibodies specific for stool-derived proteins. Results have indicated higher stool-specific IgG in the serum of P2KO as compared to that of WT mice, confirming LPS results (data not shown).

### Reduced *in vivo* Influenza Vaccine Antibody Response in P2KO vs. WT Mice

Bacterial translocation affects immune responses by inducing Immune Activation (IA) in circulating immune cells. The receptor for LPS, TLR4, is one of the several markers of IA so far identified. It is known that there is a negative association between the expression of IA markers in immune cells before stimulation and the response of the same immune cells after *in vivo* or *in vitro* stimulation. Therefore, IA is negatively associated with functional immune cells. This has been shown in chronic inflammatory conditions (aging and age-associated conditions) as well as in chronic infections (HIV, malaria) (7, 13–16). We measured *in vivo* antibody production in young and old WT and P2KO mice by measuring the serum response to the influenza vaccine by ELISA. Results in Figure 1B show that P2KO mice of both age groups have significantly decreased *in vivo* responses to the vaccine and make significantly less influenza vaccine-specific IgG antibodies as compared to WT controls. Noteworthily, the response of young P2KO mice is not different from the one observed in old WT mice. Influenza vaccine-specific IgA (Figure 1C) and total IgG show a similar pattern (Figure 1D). The influenza vaccine response, as expected, was negatively correlated with microbial translocation (Figure 1E).

### Reduced *in vitro* Class Switch in B Cells From P2KO VS. WT Mice

We then measured *in vitro* class switch, IgG secretion and plasma cell frequencies in LPS-stimulated splenic B cells from young and old WT and P2KO mice. We evaluated E47, Pax5, Prdm1 (Blimp-1), and activation-induced cytidine deaminase (AID) mRNA expression by qPCR. This was done at time points that we found optimal in our previously published work measuring *in vitro* class switch in splenic B cells from young and old C57BL/6 mice. Briefly, we found that E47 mRNA is higher at day 1 and then decreases at days 2–3 after stimulation (17, 18). Pax5 mRNA expression has a kinetic similar to E47 (unpublished). AID mRNA is already detectable at day 3 but peaks at day 5, to decrease later on (17). Prdm1(Blimp-1) is detectable at day 2 and increases at later days, peaking at day 4, and it stays up until day 7 (18).

E47 (19, 20), and Pax5 (21, 22) are transcriptional regulators of AID, the enzyme necessary for class switch recombination, the process leading to the production of secondary, class-switched antibodies, and somatic hypermutation (23–25). AID is a measure of optimal B cell function. Prdm1 (Blimp-1) is the transcription factor for plasma cell differentiation (26). In addition to transcription factors for class switch recombination and plasma cell differentiation, and AID, we also measured IgG3 secretion by ELISA. IgG3 is the Ig subclass secreted in larger amounts in response to LPS alone. In response to LPS and class switch cytokines or B lymphocyte stimulator (BlyS), a key survival factor for B cells also known to induce class switch (27), splenic B cells from 129/SvJ mice make predominantly IgG1 followed by IgG2b (28). Frequencies of plasma cells by flow cytometry were also evaluated in LPS-stimulated splenic B cells from young and old WT and P2KO mice.

**Figure 2:**
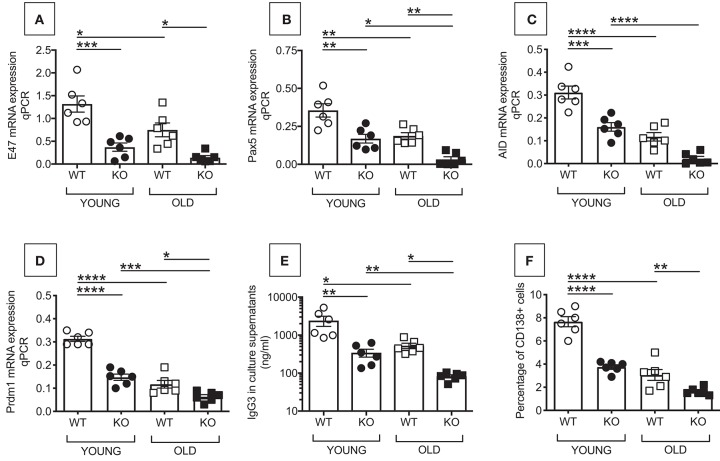
Reduced *in vitro* class switch in B cells from P2KO vs. WT mice. B cells were isolated from the spleens of young and old WT and P2KO mice (6 mice/group) by magnetic sorting. B cells (10^6^/ml) were stimulated for 1–7 days with 1 μg/ml of LPS, then mRNA was extracted and qPCR performed to evaluate mRNA expression of E47 **(A)** and Pax5 **(B)** (at day 1), AID **(C)** (at day 5), and Prdm1 (Blimp-1) **(D)** (at day 4). Results show qPCR values (2^−ΔΔCt^). At day 5, cells are harvested to evaluate frequencies of CD138+ cells by flow cytometry **(F)**. At day 7, supernatants were collected to measure IgG3 secretion by ELISA **(E)**. Mean comparisons between groups were performed by two-way ANOVA followed by Bonferroni's multiple comparisons test. *p < 0.05, **p < 0.01, ***p < 0.001, ****p < 0.0001.

Results in Figure 2 show that B cells from P2KO mice, both young and old, express significantly less mRNA for E47 (A), Pax5 (B), AID (C), and Prdm1 (Blimp-1) (D) and secrete significantly less IgG3 antibodies (E), as compared to WT controls. Also the frequencies of plasma cells are less in cultured B cells from P2KO as compared to those from WT mice (F). Again, the response of young P2KO mice is not different from the one observed in old WT mice.

### Increased Intrinsic Inflammation in Splenic B Cells From P2KO vs. WT Mice

**Figure 3:**
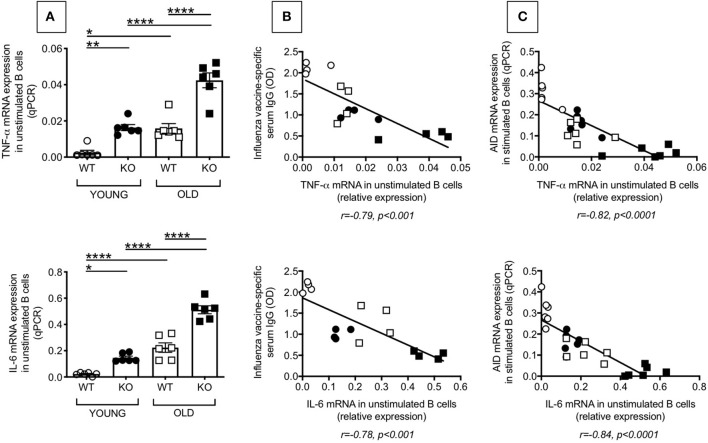
Increased intrinsic inflammation in splenic B cells from P2KO vs. WT mice. **(A)** B cells isolated from the spleens of young and old WT and P2KO mice (6 mice/group) by magnetic sorting were left unstimulated. The mRNA was extracted from the unstimulated B cells and qPCR performed to evaluate mRNA expression of TNF-α and IL-6. Results show qPCR values (2^−ΔΔCt^). Mean comparisons between groups were performed by two-way ANOVA followed by Bonferroni's multiple comparisons test *p < 0.05, **p < 0.01, ***p < 0.001, ****p < 0.0001. **(B)** Correlation of TNF-α (top) and IL-6 (bottom) mRNA expression with *in vivo* influenza vaccine antibody response. Pearson's *r* and *p*-values are at the bottom of the figure. **(C)** Correlation of TNF-α (top) and IL-6 (bottom) expression with *in vitro* class switch measured by mRNA expression of AID after 5 day stimulation with LPS. Pearson's *r* and *p*-values are at the bottom of the figure.

We have previously shown in both mice (6) and humans (7) that high TNF-α mRNA levels in resting B cells negatively correlate with the response of the same B cells when stimulated *in vivo* or *in vitro* with mitogens and/or vaccines, clearly demonstrating that the inflammatory status of the B cells impacts their own function. We therefore measured mRNA expression of the pro-inflammatory cytokines TNF-α and IL-6 in unstimulated splenic B cells from young and old WT and P2KO mice. Results in Figure 3 show that TNF-α (top) and IL-6 (bottom) mRNA expression in unstimulated B cells from from P2KO mice are significantly higher as compared to those in B cells from WT mice (A). Moreover, TNF-α (top) and IL-6 (bottom) mRNA expression in unstimulated B cells are negatively associated with the *in vivo* influenza vaccine response (B) and with the *in vitro* AID mRNA expression (C). These results altogether confirm and extend our previous findings that higher mRNA expression of the inflammatory cytokines TNF-α and IL-6 in B cells, prior to any stimulation, renders the same B cells incapable of being optimally stimulated by vaccines or mitogens.

### Increased Frequencies and Numbers of Pro-inflammatory B Cells in the Spleen of P2KO vs. WT Mice

**Figure 4:**
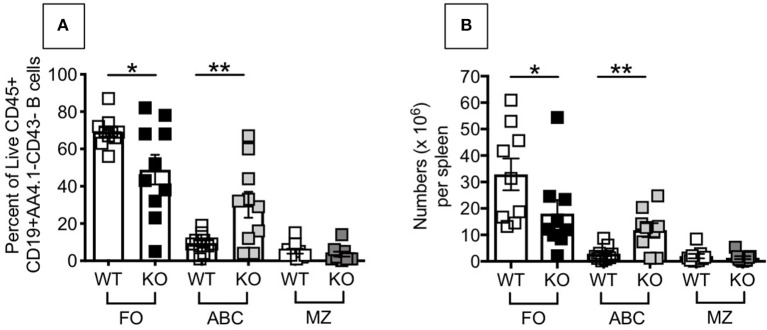
Increased frequencies and numbers of ABCs/pro-inflammatory B cells in the spleen of P2KO vs. WT mice. The spleens of old WT and P2KO mice (10 mice/group) were stained to evaluate frequencies **(A)** and numbers **(B)** of FO, ABC and MZ B cell subsets. Results are gated on Live CD45+CD19+AA4.1-CD43- cells to exclude transitional (AA4.1+) and B1 (CD43+) B cells. Mean comparisons between groups were performed by two-way ANOVA followed by Bonferroni's multiple comparisons test. *p < 0.05, **p < 0.01.

The above results, showing higher inflammation (TNF-α and IL-6 mRNA expression) in unstimulated B cells from P2KO mice, as compared to WT controls, are supported by the findings of higher frequencies of pro-inflammatory B cell subsets in the spleens of P2KO vs. WT mice, as shown in Figure 4. We previously showed in mice (29) and humans (7) conditions and pro-inflammatory B cell subsets contributing to reduced function in the aged. We measured by flow cytometry the percentages of FO, ABC and MZ B cell subsets in the spleens of WT and P2KO old mice (the ones with the highest levels of inflammation). Results show significantly reduced frequencies (A) and numbers (B) of the anti-inflammatory FO subset, and significantly increased frequencies and numbers of the pro-inflammatory ABC subset, in the spleens of old P2KO vs. WT mice. No differences in frequencies and numbers of MZ B cells were observed between WT and P2KO mice.

### No Difference Between WT and P2KO Mice in Fat Measures but Increased Frequencies of ABCs in the VAT of P2KO vs. WT Mice

Fat mass increases with age in mice (30) and humans (30, 31). The increase in fat mass with age is responsible for increased local and systemic levels of pro-inflammatory mediators that are markers of inflammaging (9). Higher fat mass also induces pro-inflammatory B cells and impairs B cell function in old mice (29) and humans (32, 33). Therefore, obesity may be considered a mechanism of aging.

**Figure 5:**
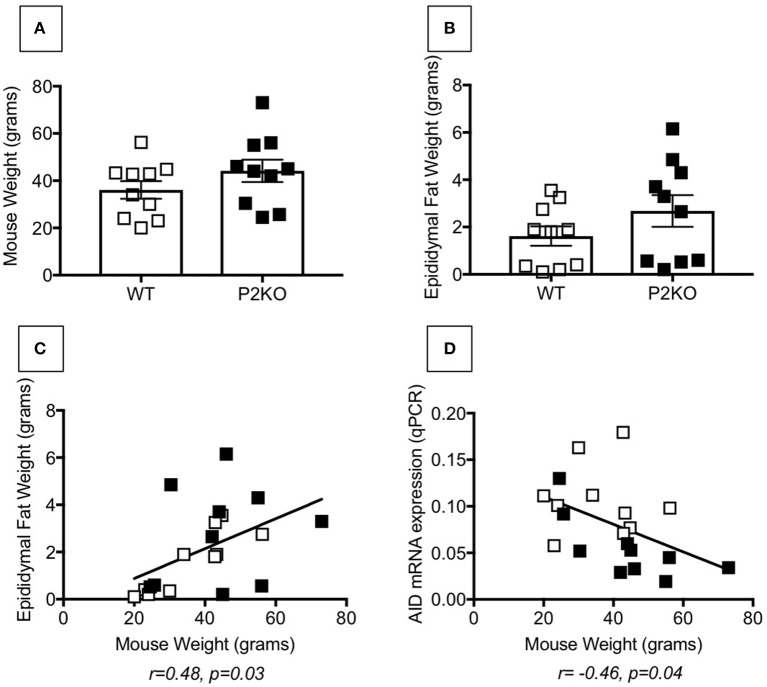
Comparable fat measures in P2KO vs. WT mice. Ten pairs of old WT and P2KO mice were sacrificed. Mouse weight and epididymal VAT weight are shown in **(A,B)**, respectively. **(C)** Correlation between mouse weight and epididymal VAT weight. Pearson's *r* and *p*-values are at the bottom of the figure. **(D)** Correlation between mouse weight and mRNA expression of AID in stimulated splenic B cells after 5 day stimulation with LPS. Pearson's *r* and *p*-values are at the bottom of the figure.

We analyzed the VAT to identify contributors to the phenotypic and functional changes observed in splenic B cells from old P2KO mice as compared to WT controls. Results in Figure 5 show that both mouse weight (A) and epididymal VAT weight (B) are comparable in WT and P2KO mice. The 2 measures are positively correlated (C). Additionally, mouse weight is negatively associated with *in vitro* class switch, measured by AID mRNA expression in stimulated splenic B cells (D).

**Figure 6:**
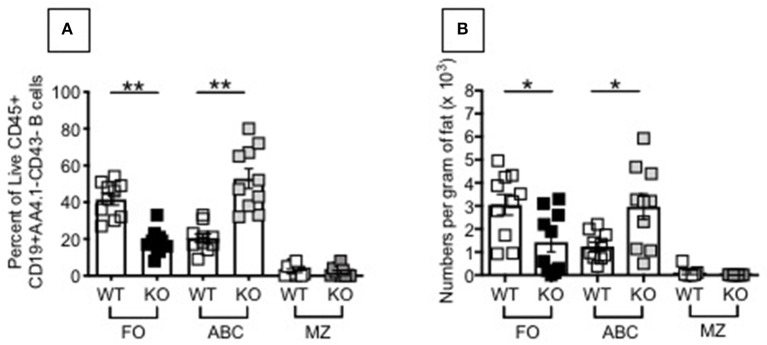
Increased frequencies and numbers of ABCs/pro-inflammatory B cell subsets in the SVF of P2KO vs. WT mice. The SVF from the VAT of old WT and P2KO mice (10 mice/group) were stained to evaluate frequencies **(A)** and numbers **(B)** of FO, ABC, and MZ B cell subsets. Results are gated as in Figure 4. Mean comparisons between groups were performed by two-way ANOVA followed by Bonferroni's multiple comparisons test. *p < 0.05, **p < 0.01.

To explain the results in D and identify mechanisms responsible for the VAT-driven inflammation leading to the down-regulation of AID, we compared frequencies of ABCs in the VAT of P2KO vs. WT old mice. Results in Figure 6 show that FO B cells significantly decrease in frequencies and numbers, while ABCs significantly increase, in the VAT of old P2KO mice as compared to age-matched WT controls. These results demonstrate that although no significant differences were observed in mouse weight and epididymal VAT weight between P2KO and WT mice, frequencies and numbers of ABCs, the most pro-inflammatory B cell subset, are increased in the VAT of P2KO mice and they contribute to local and systemic inflammation which negatively impacts B cell function.

### Increased Differentiation of ABCs in the VAT of P2KO vs. WT Mice

**Figure 7:**
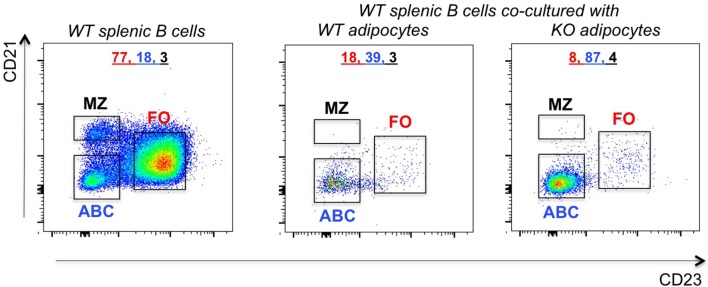
Increased frequencies of ABCs in the VAT of P2KO vs. WT mice. B cells from the spleen of old WT mice were stained as in Figure 4 and frequencies of FO, ABC, MZ were evaluated. Adipocytes were isolated from the VAT of WT and P2KO old mice and cultured for 72 h in transwells with the splenic B cells of old WT mice. After this time, B cells were stained as indicated above and the frequencies of B cell subsets measured by flow cytometry. Results are representative of four independent experiments.

To understand if ABC frequencies in the VAT of P2KO mice increase as a consequence of increased differentiation of ABCs, we performed the following experiment in which we evaluated the ability of adipocytes to induce ABCs. We co-cultured in transwells adipocytes from the VAT of WT or P2KO old mice with splenic B cells from WT mice. These experiments were performed in the absence of any exogenous stimulation. Results in Figure 7 show that co-culture of 72 hrs significantly changed the relative percentages of the B cell subsets, leading to a significant increase in ABC percentages, similar to what we have observed in the VAT (Figure 6). The reason why the co-culture of WT adipocytes and splenic B cells also changes the relative proportions of FO and ABC (reducing FO and increasing ABC percentages) is because WT adipocytes are also inflammatory, although not as much as P2KO adipocytes.

**Figure 8:**
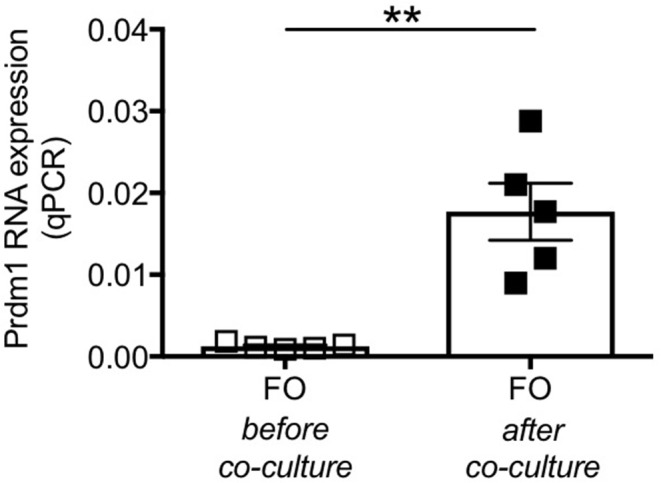
Splenic FO B cells co-cultured with P2KO adipocytes show markers of ABCs. FO B cells sorted from the spleen of P2KO old mice were co-cultured with adipocytes from the same old mice. RNA was extracted before and after 72 h in transwell and expression of Prdm1 was evaluated by qPCR. Results show qPCR values (2^−ΔΔCt^). Mean comparisons between groups were performed by paired Student's *t*-test (two-tailed). **p < 0.01.

To further confirm that FO do not decrease because they die but because they differentiate into ABCs, as we have previously shown in C57BL/6 mice (29), we co-cultured adipocytes from P2KO old mice with sorted splenic FO B cells from the same mice and we compared gene expression profiles of these FO B cells before and after 72 h in transwell. We measured Prdm1 (Blimp-1), a marker up-regulated in ABCs vs. FO, as we (29) and others (34) have previously shown. Results in Figure 8 show that the co-culture with adipocytes induced differentiation of FO B cells into ABCs, as splenic FO B cells acquired markers typical of ABCs. It is relevant to note that adipocyte-driven ABC differentiation occurred in the absence of any exogenous (antigen/mitogen) stimulation. This culture condition is different from that in which FO are stimulated with antigens/mitogens *in vitro* to generate Prdm1 (Blimp-1) expressing plasma cells.

### Adipocytes From P2KO Mice Are More Inflammatory Than Those From WT Mice

**Figure 9:**
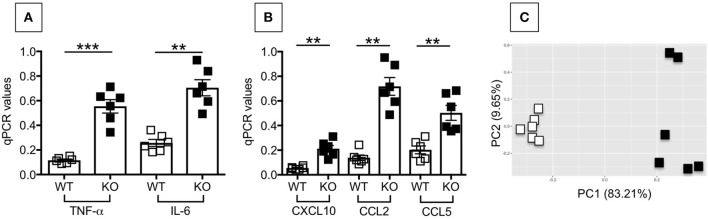
Adipocytes from P2KO mice are more inflammatory than those from WT mice. Adipocytes were isolated from the VAT of WT and P2KO old mice, sonicated for cell disruption in the presence of TRIzol to separate the soluble fraction from lipids and cell debris. Results show qPCR values (2^−ΔΔCt^) of TNF-α and IL-6 **(A)**, CXCL10, CCL2, and CCL5 **(B)** RNA expression. Mean comparisons between groups were performed by Student's *t*-test (two-tailed). **p < 0.01, ***p < 0.001. **(C)** PCA analysis with the axes showing the percentage of variation explained by PC1 and PC2. Each symbol corresponds to one mouse.

We then compared the inflammatory profile of adipocytes from the VAT of WT and P2KO mice, which is responsible for the recruitment of inflammatory B cell subsets to the VAT and for their differentiation. We measured in particular RNA expression of pro-inflammatory cytokines (TNF-α, IL-6) and chemokines (CXCL10, CCL2, CCL5). Results in Figure 9 show significantly higher expression levels of the RNA for pro-inflammatory cytokines (A) and chemokines (B) in adipocytes from P2KO mice as compared to WT controls. In the PCA analysis (C) we show distinct clustering of the 2 groups of adipocytes.

## Discussion

The mouse and human gastrointestinal tracts are colonized by a huge number of microorganisms. Although the gut provides a functional barrier between these microorganisms and the host, translocation of bacteria and/or their products is still occurring even in normal, healthy conditions. Our study is based on the hypothesis that these events of microbial translocation are strongly suspected to lead to the establishment of systemic chronic inflammation, intrinsic B cell inflammation and dysfunctional antibody responses. P2KO mice, lacking the mechanisms to control the proliferation and dissemination of the different microbes, are characterized by higher intrinsic B cell inflammation and more dysfunctional antibody responses as compared to WT controls. This is clearly shown by increased microbial translocation in the serum of P2KO mice as compared to WT controls, which is negatively associated with a protective response against the influenza vaccine. This is to our knowledge the first study evaluating B cell function and antibody responses in mice lacking P2.

Studies in mice have clearly demonstrated that intestinal components also regulate the VAT [reviewed in Tilg and Kaser (35)] and results have shown that gut permeability is increased in obesity (36, 37) leading to the release of LPS in the circulation. LPS, as well as other intestinal antigens, has been shown to be absorbed in the VAT through lipid-driven mechanisms (38, 39).

Based on our previous data in aged mice and humans, we know that the inflammatory status of the individual and of B cells themselves impacts B cell function. Here we show that the ability to generate an *in vivo* specific antibody response to the influenza vaccine is reduced in P2KO mice as compared to WT controls. The class switch recombination defects at least in part responsible for the reduced *in vivo* and *in vitro* antibody responses include the reduced expression of AID and of its transcriptional activators E47 and Pax5. Moreover, splenic unstimulated B cells from P2KO mice make higher levels of TNF-α and IL-6 mRNA than those from WT mice and these negatively correlate with B cell function, measured *in vivo* by the response to the influenza vaccine and *in vitro* by AID mRNA expression in stimulated B cell cultures. These results confirm and extend our previously published results showing a negative impact of systemic chronic inflammation on B cell function and antibody production *in vivo* and *in vitro*.

This inflammatory status of the splenic B cells is associated with increased frequencies and numbers of the pro-inflammatory B cell subset called ABCs. These cells have been reported to increase in aging and in age-associated inflammatory conditions in both mice (29, 34, 40, 41) and humans (42–46). These cells have a unique transcriptomic phenotype (34) and are characterized by a senescence-associated secretory phenotype responsible for the secretion of several pro-inflammatory markers, including chemokines, cytokines, growth factors and matrix metalloproteinases (47).

ABCs not only increase in the spleens but also in the SVF of the VAT of P2KO mice, despite a lack of increase in mouse weight and fat mass. The reason for us to evaluate the VAT is because with aging the VAT undergoes significant changes in abundance, distribution, cellular composition, endocrine signaling and it has been shown to affect the function of other systems including the immune system. ABCs differentiate in the VAT following interaction with the adipocytes and this occurs more in the VAT of P2KO mice as compared to WT controls. Differentiation of ABCs in the VAT is accompanied by the acquisition of markers typical of this B cell subset and expressed at almost indiscernible levels in FO B cells. We measured Prdm1, the gene coding for Blimp-1, the transcription factor for plasma cells, among others, as the RNA expression of this marker was found 10-fold higher in unstimulated splenic ABCs vs. FO in our previously published study (29). Although the major function of adipocytes is to store excess energy, several recent findings have indicated that the adipocytes are also endocrine cells able to secrete adipokines and several pro-inflammatory molecules that modulate immune cell infiltration, immune cell activation and differentiation. We have preliminary evidence that leptin, the major adipokine secreted by the adipocytes, induces *in vitro* differentiation of splenic naïve B cells into ABCs secreting IgG2c autoantibodies (data not shown). Experiments currently under way in our laboratory are evaluating other adipocyte-derived molecules that may be involved in B cell differentiation in the VAT.

In conclusion, our results show for the first time that P2KO mice have decreased antibody responses, likely consequent to changes in B cell characteristics/function, chronic systemic inflammation supported by a continuous microbial translocation from the gut which cannot be controlled as these mice lack P2. These results are physiologically relevant for patients, although not frequent, with P2 deficiency who contract infections with intracellular bacteria (48) and therefore may need to be treated to improve their humoral immunity.

## Data Availability Statement

The data generated in this study are available upon request to the corresponding author.

## Ethics Statement

The animal study was reviewed and approved by University of Miami IACUC approved protocols #16-252 and #16-006.

## Author Contributions

DF and BB conceived the experiments. DF coordinated the experiments. DF, AD, MR, and TV performed the experiments and analyzed data. DF and AD performed statistical analyses. RM, LR, and NS provided reagents. DF wrote the paper. All authors were involved in writing and had final approval of the submitted and published versions of the paper.

### Conflict of Interest

RM and EP are inventors of patents and stand to gain royalties from future commercialization. The remaining authors declare that the research was conducted in the absence of any commercial or financial relationships that could be construed as a potential conflict of interest.

## Dedication

This paper is dedicated to the memory of Eckhard R. Podack.
